# Water and wisdom: Hydration as a defence against dementia

**DOI:** 10.1113/EP093168

**Published:** 2025-09-02

**Authors:** David C. Byfield, Damian M. Bailey

**Affiliations:** ^1^ Neurovascular Research Laboratory, Faculty of Life Sciences and Education University of South Wales Pontypridd UK; ^2^ Bexorg, Inc. New Haven Connecticut USA

With 55 million people currently affected worldwide and projections estimating a rise to 153 million by 2050, dementia presents a growing global health crisis (GBD 2019 Dementia Forecasting Collaborators, 2022; Livingston et al., 2024). Furthermore, cognitive impairment, the preclinical and transitional stage of dementia, affects an estimated 16%–20% of adults aged over 50 years, underscoring the scale of this disease (Bai et al., 2022; Salari et al., 2025).

Modifiable risk factors, such as physical inactivity, hypertension, dyslipidaemia, diabetes, smoking and alcohol misuse, dominate the dementia prevention discourse (Livingston et al., 2024). However, hydration status remains largely overlooked despite growing physiological and epidemiological evidence suggesting that chronic hypohydration might contribute significantly to cognitive decline (Dmitrieva et al., 2024; Nishi et al., 2023). Dehydration (serum osmolality >300 mOsmol/kg H_2_O is particularly prevalent in older adults, often owing to impaired thirst perception, renal dysfunction, polypharmacy, cognitive deficits, communication barriers, mobility limitations and dependence on caregivers (Hooper et al., 2016; Parkinson et al., 2023).

Dehydration affects an estimated 24% of non‐hospitalized older adults (Parkinson et al., 2023), with prevalence reaching 20% in long‐term care residents (DRIE Study; Bunn & Hooper, 2019) and ≤50% in those with cognitive impairment (Aslan Kirazoglu et al., 2024); figures that far exceed rates in cognitively intact populations (Stookey, 2005). These bidirectional relationships raise clinical concern; dehydration can exacerbate cognitive decline, and cognitive impairment itself increases vulnerability to dehydration (Hooper et al., 2014).

Approximately 75% of brain tissue is water, and even mild dehydration (equivalent to 1%–2% body water loss) impairs mood, working memory and executive function (Adan, 2012; Masento et al., 2014). Although acute cognitive effects are documented, the chronic consequences of low fluid intake are less understood. Emerging data link habitual hypohydration to reduced brain volume and diminished cognitive performance in older adults (Wittbrodt & Millard‐Stafford, 2018), suggesting potential cumulative harm.

Dehydration adversely impacts cerebrovascular function, reducing cerebral blood flow and impeding the corresponding delivery of oxygen and glucose, which are essential substrates for brain metabolism (Ogoh & Ainslie, 2009). Depressed bioenergetic function can increase susceptibility to neuronal injury, particularly in ageing brains (Pross, 2017). Mechanistically, dehydration leads to haemoconcentration, increased blood viscosity and reduced cerebral perfusion pressure (Perry et al., 2016; Trangmar et al., 2015), potentially accelerating the formation of white matter lesions and small vessel disease, which are key contributors to vascular cognitive impairment (Iadecola, 2013; Rajeev et al., 2023).

Moreover, altered electrolyte homeostasis disrupts synaptic and neuronal function (Jacoby, 2020; Poe et al., 2024; Popkin et al., 2010), while dehydration‐induced hyperosmolality promotes vasopressin release, compounding vasoconstriction and vascular endothelial dysfunction that further compromise the integrated regulation of cerebral bioenergetic function (Faraco et al., 2014). Systemic oxidative–inflammatory–nitrosative stress is a shared feature of dehydration and neurodegeneration, with free radicals and associated reactive oxygen/nitrogen species thermodynamically poised to disrupt the structural integrity of the neurovascular unit (Bailey et al., 2022). Additionally, dehydration impairs glymphatic clearance of neurotoxins, such as β‐amyloid, potentially accelerating the neuropathological progression of Alzheimer's disease (Iliff et al., 2012; Kylkilahti et al., 2021).

Despite these emergent findings, hydration remains largely absent from dementia prevention strategies and is rarely included in public health guidance or clinical risk assessments (Higgins et al., 2023; Li et al., 2023; Wilson & Dewing, 2020). Given its physiological impact on oxidative–inflammatory–nitrosative stress, bioenergetic and glymphatic function, hydration status should be prioritized as a modifiable determinant of overall brain health.

Routine hydration assessment in older adults (especially during illness, heat stress or hospitalization) is essential. Interventions such as personalized hydration plans, staff education and fluid monitoring in care settings can reduce the prevalence of dehydration (Hooper et al., 2015). Moreover, integrating hydration into cognitive risk models could enhance early prevention strategies (Nishi et al., 2023).

The physiological community needs to recognize that dehydration represents a serious yet under‐recognized threat to cerebrovascular and cognitive health in ageing populations. Its correction is straightforward, cost‐effective and, potentially, protective against progression of dementia. Future research should focus on understanding the long‐term impact of hydration on brain structure and function, the identification of optimal hydration thresholds to enhance cognitive resilience, and interventions aimed at preserving brain health through individually optimized hydration. Although traditionally taken for granted, hydration might hold untapped physiological potential for safeguarding the ageing brain and must be recognized as central to both systemic and neurological homeostasis.

## CONFLICT OF INTEREST

D.M.B. is Editor‐in‐Chief of Experimental Physiology and outgoing Chair of the Life Sciences Working Group and outgoing member of the Human Spaceflight and Exploration Science Advisory Committee to ESA. D.M.B. is a current member of the ESA‐HRE‐Biology Panel and Space Exploration Advisory Committees to the UK and Swedish National Space Agencies and member of the National Cardiovascular Network for Wales and South‐East Wales Vascular Network.

## FUNDING INFORMATION

D.M.B. is supported by a Royal Society Wolfson Research Fellowship (Grant No. WM170007).

[Correction made on 9th September 2025, after first online publication: Conflict of interest and funding information sections have been updated.]
